# Leader evaluation and team cohesiveness in the process of team development: A matter of gender?

**DOI:** 10.1371/journal.pone.0186045

**Published:** 2017-10-23

**Authors:** Núria Rovira-Asenjo, Agnieszka Pietraszkiewicz, Sabine Sczesny, Tània Gumí, Roger Guimerà, Marta Sales-Pardo

**Affiliations:** 1 Department of Chemical Engineering, Universitat Rovira i Virgili, Tarragona, Catalonia, Spain; 2 Department of Psychology, Division of Social Psychology and Social Neuroscience, University of Bern, Bern, Switzerland; 3 Institució Catalana de Recerca i Estudis Avançats (ICREA), Barcelona, Catalonia, Spain; Northwestern University, UNITED STATES

## Abstract

Leadership positions are still stereotyped as masculine, especially in male-dominated fields (e.g., engineering). So how do gender stereotypes affect the evaluation of leaders and team cohesiveness in the process of team development? In our study participants worked in 45 small teams (4–5 members). Each team was headed by either a female or male leader, so that 45 leaders (33% women) supervised 258 team members (39% women). Over a period of nine months, the teams developed specific engineering projects as part of their professional undergraduate training. We examined leaders’ self-evaluation, their evaluation by team members, and team cohesiveness at two points of time (month three and month nine, the final month of the collaboration). While we did not find any gender differences in leaders’ self-evaluation at the beginning, female leaders evaluated themselves more favorably than men at the end of the projects. Moreover, female leaders were evaluated more favorably than male leaders at the beginning of the project, but the evaluation by team members did not differ at the end of the projects. Finally, we found a tendency for female leaders to build more cohesive teams than male leaders.

## Introduction

The impact of leader gender on the evaluation of leaders has been studied for quite some time (e.g., [1,2]). While researchers agree that there are negligible gender differences in leaders’ performance [3], the debate on whether gender influences leader’s self-evaluations and their evaluation by others is still on-going [4–6]. Past research using experimental laboratory studies and organizational studies has documented that female leaders are equally effective as male leaders [3,7], yet female leaders are evaluated slightly less favorably than male leaders (e.g., [1,8,9]).

One of the reasons why female leaders receive less positive evaluations is gender stereotyping: women and men are perceived differently in the context of leadership (e.g.,[1–5]). Women in Western societies are generally seen as more communal (e.g., caring, emotional, helpful) than men, who are perceived as more agentic (e.g., active, decisive, dominant) (e.g., [6,7]). The stereotype of leaders is closer to the male stereotype, that is, more agentic than communal (see [8] for an overview), documented in numerous studies with different methodologies (e.g., [4]) and different sample populations (e.g., [9]). The mismatch between female stereotype and leader role ([1,10]) also has significant consequences for women’s self-views. Women see themselves as less agentic and more communal than men (e.g., [11,12]) and as less suited for a leadership position [13]. In contrast to evaluations by others, female leaders tend to consider themselves less effective than male leaders [14].

The present research aims to determine the impact of gender stereotypes on leader evaluation in a real-life process of team development, focusing on the following questions: (1) Do gender stereotypes influence the self-evaluations of leaders? (2) Do gender stereotypes influence the way female and male leaders are evaluated by others? If so, does the impact of gender stereotypes on self-evaluations and evaluations by others remain stable or does it change in the process of team development, as increasing experience with the respective individual leads to a decrease in stereotyping? In addition, we are interested in the structure of the networks that develop around female and male leaders: (3) To what extent does a leader’s gender affect social relationships within the team, specifically team cohesiveness? In dealing with these questions, the present research extends past findings in at least three distinct ways:

First, the present study assesses the impact of gender stereotypes on leader evaluation in *a real-life team building process*. The majority of earlier findings are based on laboratory settings (e.g., [15–18]). Only few studies have been conducted in organizational contexts (e.g., [19]), even though research settings are known to have a significant impact on leader evaluation (see [20] for a review). Since increased exposure to a particular person reduces the stereotyping of that person [21], studies based on hypothetical or laboratory leaders may yield stronger gender differences than studies on real-life superiors in actual organizations [19]. The present study therefore investigates leaders and team members that were involved in real projects as part of their academic curriculum. The project outcomes actually affected their academic records, a factor which is absent in laboratory settings. Thus, leader evaluation within this professional setting had ecological validity and can help to understand leader evaluation during team development.

Second, our study examines *longitudinal changes*, both in the self-evaluation of leaders and in the evaluation by members of their teams. The aim is to assess whether the impact of gender stereotypes remains stable or changes during team development. Although gender stereotypes are deeply rooted in society and therefore difficult to overcome [7,22], previous research has documented that increased exposure to an individual results in a decrease of stereotyping (e.g., [21,23]). During impression formation people simplify their evaluation of others by classifying them as members of certain groups (e.g., female individuals are automatically associated with the group of women and male individuals with the group of men, as individuating requires too much mental effort; see [21,24]). As a result, first reactions to individuals are based on stereotypes (e.g., [25,26]). If sufficient time and/or mental resources become available, however, individuation, takes place. Now perceivers base their impressions on attributes of the individuals they have become acquainted with and modify their initial judgments (e.g., [27]). In this way, the influence of gender stereotypes on the evaluation of women and men in leadership positions may decrease over time. To the best of our knowledge, the present study is the first piece of research addressing this question.

Finally, our study extends the present debate on the *role of gender for the functioning of the team*. The existing literature shows that various tasks (e.g., management or research) are increasingly accomplished by groups [28,29], hence it is important to examine the effect of gender on team processes. Earlier studies on gender differences in leadership have mainly focused on members’ evaluations of team leaders (e.g., [16,19]). Our study extends such investigations by examining the quality of relations between leaders and team members (i.e., the structure of teams headed by female and male leaders). Specifically, we determine team cohesiveness, that is, the density of connections between team members, and the clustering of connections around leaders (the formation of triangles of connections within the team that involve the leader). In this way, we assess whether leader gender affects the network structure of teams. So far, there is little research on leadership and social networks [30–32]. But the use of network measures to capture leadership dynamics is a growing field which lends itself to research with an additional gender perspective. The present study is the first that investigates the relationship between leader gender and team cohesiveness, and can therefore contribute to on-going debates on the role of gender in team performance (e.g., [33–35]).

### Leaders’ self-evaluation and their evaluation by team members

Earlier research has shown that gender stereotypes may limit women’s *self-evaluation*, for instance, their confidence in succeeding at male-typed tasks and occupations (e.g., [36,37]). This may damage their performance or their career aspirations (e.g., [38,39]). For instance, female students were found to underestimate their competence in performing a scientific task in comparison to male students [40]. Also, male leaders tended to overestimate their effectiveness, while female leaders perceived themselves to be at the same level as their subordinates [41,42]. This may be explained by the fact that men tend to have higher self-esteem than women (e.g., [43]) and self-esteem is predictive for how people evaluate their own performance [44,45]. However, leaders’ self-evaluation may change during team development: Past research suggests that in organizational settings women gain self-confidence or self-esteem in achievement situations (e.g., [46,47]). Thus, female leaders’ self-appraisal may come to be less affected by gender stereotypes in the process of team development and they may evaluate themselves more favorably over time.

#### Hypothesis 1

We assume that, due to the impact of gender stereotypes on women’s self-views [40], female leaders evaluate themselves less favorably than male leaders at the beginning of the project (Time I); this gender difference will have disappeared at the end of the project (Time II).

Gender stereotypes also affect the *evaluation by others*. Due to the mismatch between traditional conceptions of leadership and the female stereotype there is a tendency to judge women as less suitable leaders than men [1,8,10,48,49]. Furthermore, female leaders tend to be evaluated less favorably than male leaders (e.g., [50]). The influence of gender stereotypes is especially important in impression formation. Thus, the less time respondents had to read a vignette about hypothetical leaders, the more favorable were their evaluations of male compared to female leaders [51]. Paradoxically, a recent meta-analysis of 95 studies documents that basically female leaders seem to be evaluated as more effective than male leaders [14]. The shifting standards model [52,53] offers an explanation for such favorable evaluations of female leaders. This model posits that people judge members of groups by category-specific standards, that is, they compare them to specific within-category standards. According to gender stereotypes, people judge male leaders as more capable than female leaders (e.g., [54,55]. Consequently, they tend to evaluate the leadership competence of a particular woman in relation to women’s supposedly lower standards of competence, and the leadership competence of a particular man in relation to men’s supposedly higher standards of competence [56]. As a result, a woman assigned as leader may be evaluated more favorably (due to the lower standards applied) than a man with equal competences in the same situation (due to higher standards). Based on the recent meta-analytical findings [14], it is reasonable to assume that team members’ evaluations of female leaders surpass evaluations of men at the beginning of the project, especially in a male-dominated field such as engineering as in the present research. That is, at the beginning of the projects female leaders may be evaluated based on their group membership (i.e., as tokens). Over time, however, as team members become increasingly familiar with their individual leader, female leaders may no longer benefit from within-category standards. Instead, team members may base their evaluations on between-category standards.

#### Hypothesis 2

We expect team members in a male-dominated field (engineering) to evaluate female leaders more favorably than male leaders at the beginning of the teamwork (Time I), due to within-category judgment [57,58]. This gender difference is assumed to disappear towards the end of the project (Time II) as a result of individuation [23,26,59].

### Cohesiveness in the process of team development

Cohesiveness is “the degree to which members are attracted to a group and motivated to remain part of it” [35]. Cohesiveness was found to be associated with, among other things, a decrease in team conflicts [60,61], innovation in teams [62], and improvement in individual performance [63,64]. Thus, informal connections between individuals can have important implications for the team as a whole, as they limit or facilitate the flow of resources between and within teams [65]. A meta-analysis of 37 studies documents that teams with dense networks of interpersonal ties are more successful in achieving their goals and are more motivated to stay together [66]. Nonetheless, the relationship between the density of connections within a team and team performance is still unclear: while some scholars found that the density of the network of informal social ties was positively associated with team performance [67,68], other studies did not confirm these findings [69].

Numerous authors have claimed that effective leadership combines the ability to “accurately perceive the network relations that connect people, and to actively manage these network relations” [70]. Recent findings have indeed documented an impact of network relationships on team effectiveness (e.g., [66]). Recent research by Post [71] indicates that teams with female leaders are more cohesive and more cooperative than teams with male leaders. This may be due to gender differences in other-orientation, as women were found to be more empathic (e.g., [72–74]), more supportive [75,76], more cooperative, collaborative, more oriented towards enhancing others’ self-worth, and at the same time less hierarchically oriented than men [77–79]. Furthermore, female leaders are commonly expected to devote themselves to the common good of a team or a company in [5], an expectation which may contribute to an increased team cohesiveness in female-led teams. Therefore, it is plausible to assume that networks will become more cohesive around female leaders in the process of team development.

In our study, we followed the approach of Quintane and colleagues [80], who argued that there are two aspects that contribute to team cohesiveness: reciprocity in social interactions and closure (in other words, the presence of triangles of reciprocal interactions). Our analysis thus focuses on two factors: the cohesiveness of the network of connections within the team and the relative cohesiveness of the network of connections around the leader. Analysis of the former factor makes our study comparable to that of earlier analyses. Analysis of the latter factor enables us to exploit information on how network connections arrange themselves around the leader and to assess structural differences that result from leadership over time, particularly differences in the structural role of leaders. Analyzing the teams’ networks also enables us to go beyond previous studies by assessing whether changes in evaluations are related to changes in the social structure of a team, specifically to changes in the role of the leader. Considering these aspects, we predict the following:

#### Hypothesis 3

Overall team cohesiveness and relative cohesiveness around the leader, as defined by the team’s network of social interactions, do not differ for female- and male-led teams at the beginning of the projects (Time I). However, female-led teams become more cohesive around the leader than male led-teams towards the end of the projects (Time II) (see [71]).

## Methods

The data used in the study is exempt from Institutional Review Board review because of the following reasons: i) In the study we do not collect the data ourselves but we use existing data—team members-evaluations (i.e., other-evaluations and network data) and self-evaluations of team leaders (i.e., self-evaluations) that were routinely collected as part of the evaluation process of the Integrated Project (first year mandatory course) and the Team Leadership and Management (senior year course) since 2008 (our data is from 2010 to 2013) at the Universitat Rovira i Virgili, Spain. Students provided consent to participate in the courses mentioned before and to use this data for educational and research purposes; ii). The data was collected by the university that gave us access to anonymized version of the data. Students cannot be identified, neither directly nor through identifiers linked to them. Their names are anonymized, and we have no demographic information other than gender. We do not have access to any information that could identify individual participants during or after data collection.

Our sample consists of engineering students who were involved in projects at their university. The purpose of these projects was to have the students apply the knowledge and skills acquired in different courses to solve an engineering problem (e.g., the pre-design of a chemical plant) over the course of nine consecutive months. The projects were headed by senior-year students who had been carefully selected for this program (see below). In the framework of the program, team members met with their leaders on a weekly basis to discuss the progress of their work, their achievements and future steps.

As an integral part of project evaluation, team members (including leaders) evaluated other team members and provided self-evaluation in an online survey that was administered twice. Collaboration of the teams always started in September. The first measurement (Time I) took place three months later in December. The second evaluation (Time II) took place in the ninth and final month of the collaboration. The evaluations were part of the course syllabus. Part of this data has already been used for other research purposes, namely for the prediction of conflicts in teams (see [81]).

The list of abbreviations used in the study is presented in Table 1.

**Table 1:** Table of abbreviations used in the manuscript.

Abbreviation	Description
ANOVA	Analysis of Variance
*C*	Clustering coefficient
DP	D’Agostino and Pearson normality test statistic
*F*	*F*-ratio statistic (used in ANOVA)
ICC	Intra-class correlation coefficients
*M*	Mean
*Mdn*	Median
*N*	Sample size
*p*	Probability
*r*	Pearson's correlation coefficient
*RC*	Relative clustering coefficient
r_WG(j)_	Inter-rater agreement index
*SD*	Standard error
*t*	*t*-test statistic
*U*	Mann-Whitney test statistic
*Z*	Wilcoxon signed-rank test statistic
η^2^	Measure of effect size (used in ANOVA)
*ρ*	Spearman’s rank correlation coefficient

### Sample

The sample includes 45 small teams consisting of 3 to 7 student members; most teams had 5–6 members. In total, we analyzed data of 303 individuals, 45 of whom were team leaders (15 females; 30 males) and 258 team members (100 females; 158 males). The average proportion of females per team was 39%. In our sample, the gender composition of teams varied widely (proportion of females ranging from 0% to 75%). Although our sample was too small to investigate the effect of gender composition in a systematic and conclusive manner, we argue that gender composition is unlikely to have affected our findings to a large extent, because (i) we did not find significant differences between the gender composition of male- and female-led teams (*U*_*female*_ = 195.5; *p* = .30); (ii) the overall team composition is rather balanced within both male and female headed teams (*Mdn* = .5).

Team members were first-year students of chemical engineering at a public Catalan university, enrolled in a mandatory course of their academic curriculum, while leaders were senior-year students enrolled in a course on project management. In order to increase chances of success, the senior students underwent a selection process to become team leaders. All teams worked on similar projects. The participants did not know each other at the start of the project.

### Design

In the study, we measured the self-evaluation and other-evaluation of leaders at two stages of team development: at the beginning (Time I) and at the end of team development (Time II). The data was collected via online surveys over three academic years (the project teams worked together during one of the following academic years: 2010–2011, 2011–2012, and 2012–2013). Note that the overall evaluations of leaders were measured only in the last two academic years, hence the data set for this particular variable is smaller (*N* = 29; 11 female leaders; 18 male leaders). All participants logged into a specially designed online platform and completed the survey individually. They were not paid for participation. The raw data is available in the Supporting Information (S1 Dataset).

### Leader evaluation measures

Leadership requires various specific skills such as coordination, time control, conflict management, or motivating team members to achieve objectives [82]. Therefore in the present study, leaders were asked to evaluate themselves regarding four aspects: “Please evaluate the following aspects of yourself: (1) management skills (coordination, task management, time control, etc.), (2) moderation skills (securing participation of all members, conflict management, public recognition of good work, etc.), (3) motivational skills (motivation of team members, stimulating change in team members’ behavior to achieve objectives, etc.), and (4) empathy with team members.” Responses to each item were given on 11-point rating scales (0 = non-existent/poor, 10 = completely developed/excellent). We averaged individual responses for each leader and created self-evaluation indices (SE) for Time I and Time II (Cronbach’s alpha = .69 and .78 respectively). A factor analysis (principal axis) using direct oblimin rotation (see [83]) revealed that all items of the respective dimensions loaded sufficiently on the first factor (i.e., > .30), both for Time I and Time II.

We assessed the evaluation of leaders by team members regarding the same four aspects (OE). The instruction was as follows “Please evaluate the following aspects of *name of leader*”. Responses were again marked on 11-point rating scales (0 = non-existent/poor, 10 = completely developed/excellent). Subsequently, we computed the average evaluation by team members for each leader. Then we performed two principal factor analyses on this evaluation, one for Time I and another for Time II. The factor analysis showed that all items loaded sufficiently on one factor (i.e., > .30) for both points of time. We then created two reliable indices of other-evaluations of leaders (OE) for Time I and II (Cronbach’s alpha .95 and .90, respectively). In addition, the participants gave an overall evaluation of their leader (OOE). The question was as follows: “What is your overall evaluation of your leader?” Answers were given on an 11-point scale (0 = poor; 10 = excellent).

Taken together, we had one measure for the self-evaluation of leaders: the Self-Evaluation Index (SE), and two measures for the other-evaluation of leaders: the Other-Evaluation Index (OE) and the Overall Other-Evaluation Index (OOE).

#### Internal consistency of teams

To determine if team-level aggregation of our dependent variables was empirically justifiable, we took two precautions. First, we calculated within-group agreement (r_WG(j)_; [84]). Specifically, we computed the r_WG(j)_’s using the evaluations at Time I and Time II for two cases: (a) assuming an expected uniform distribution of ratings between 0 and 10; and (b) performing a random group resampling for each team [85]. For case (a) we found that for all teams r_WG(j)_’s fall within the desired range (*Mdn* = .985). For case (b) we found that for 40 out of the 45 teams r_WG(j)_’s were within the desired range (*Mdn* = .917). The other five teams had consistently lower-scoring leaders. In these teams, members consistently gave lower scores than in other teams but the ratings displayed a larger variability, therefore we did not exclude these teams from our analysis.

Second, we performed intra-class correlation coefficients tests [86]. For other-evaluation (OE) ICC(1) was .719, *F*(43,43) = 3.55; *p* < .0001. Although no absolute standard value for aggregation based on r_WG(j)_ and ICC has been established, an r_WG(j)_ equal to or greater than .70 and ICC(1) values exceeding .05 [85] are considered sufficient to warrant aggregation. Based on these indicators, we aggregated the individual-level dependent variable measures to team-level variables [85].

#### Network construction

In the survey, each team member (including the leader) answered one yes/no question for every other team member: “Would you choose this person to work with you in a new team?” We used the answers to this question to construct the network of relationships within each team at Time I and Time II. Specifically, we constructed directed team networks for Times I and II. If team member A answered “yes” when asked about team member B, we drew an edge from A to B; otherwise, we did not. This question served as a measure of team structure.

*Notes on the statistical analysis*. Due to the small size of our sample, we paid particular attention to the statistical treatment of this data, especially to meeting the requirements for analyses of variance (ANOVA) or *t*-tests for related samples for sample comparison. To that end, we applied the D’Agostino and Pearson (DP) normality test [87] to our samples. For overall other evaluation (OOE) we found that for female OOE at Time II and female OOE change did not pass the DP normality test (*p* < .01). For the Other-Evaluation Index (OE), we found that female OEs at Times I and II and male OEs at Time II were non-Gaussian (DP, *p* < .01). All samples for self-reported evaluations passed the normality test. Finally, none of the samples for the network analysis passed the normality test. Therefore, as tests involving non-Gaussian samples we used the Mann-Whitney U test for independent samples as the appropriate non-parametric test instead of ANOVA, and the Wilcoxon signed-rank test for matched samples instead of the *t*-test for related samples. In any case, our results are fairly independent of the tests we used. In order to analyze the correlations between variables, we first set up scatter plots for each pair of variables, most of which showed linear trends. We therefore tested the significance of the linear correlations between different variables by computing Pearson’s *r* and obtaining its corresponding *p*-value. The results we report do not change if we compute the non-parametric Spearman’s ρ.

## Results

### Evaluations of female and male leaders

First, we analyzed the *self-evaluations of leaders (SE)*. We included the self-evaluations of all leaders who answered the questionnaire at both times of measurement (*N* = 36; 14 female leaders; 22 male leaders). We conducted a 2 (gender of leader: female vs. male) x 2 (time: Time I vs. Time II) analysis of variance, with SE as dependent variable. Gender of leader was a between-subjects variable, time a within-subjects variable. This analysis revealed a significant main effect of time, *F*(1,34) = 10.112, *p* = .003, η^2^ = .229: leaders evaluated themselves more favorably at Time II (*M* = 8.33, *SD* = .80) than at Time I (*M* = 7.94, *SD* = .85). The interaction between gender of leader and time did not reach the conventional level of significance, *F*(1,34) = 3.01, *p* = .092, η^2^ = .081. However, we found that female leaders evaluated themselves significantly better at the end of the project (*M* = 8.71, *SD* = .94) than at the beginning (*M* = 8.02, *SD* = .90), *t*(13) = -2.32, *p* = .037 (see Hypothesis 1), whereas male leaders’ self-evaluations did not change over the course of the project, *t*(21) < 1. Moreover, female leaders evaluated themselves more favorably at Time II than male leaders did (*M* = 8.71, *SD* = .94 vs. *M* = 8.1, *SD* = .69), *t*(36) = 2.45, *p* = .019. For Time I, there was no significant difference, *t*(36) < 1 (see Hypothesis 1).

**Fig 1:**
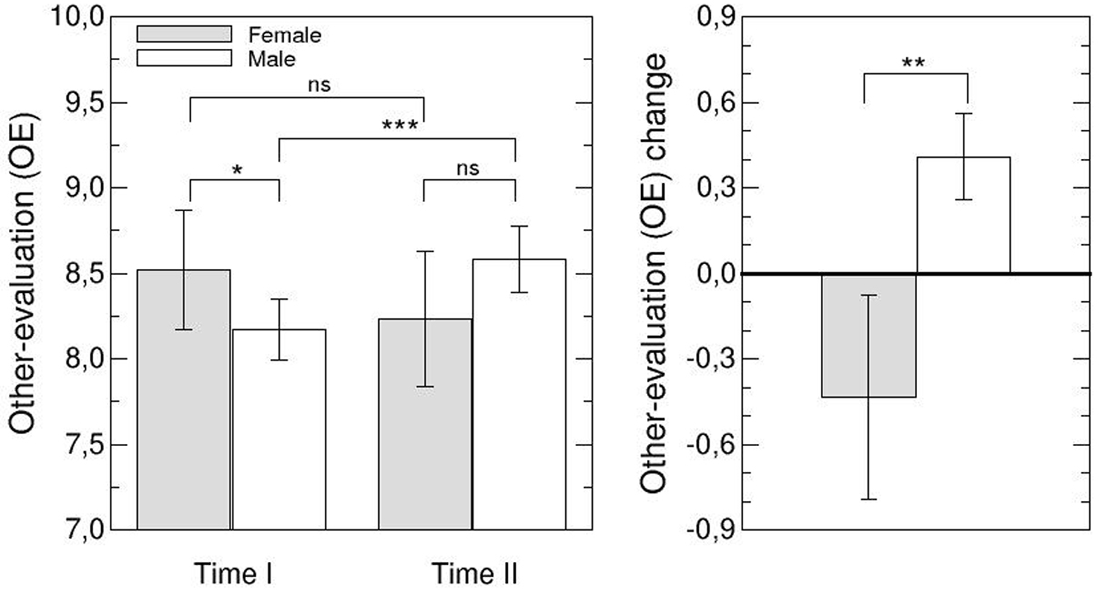
Analysis of other-evaluation index (OE). The left plot shows mean OEs at Time I and Time II for male and female leaders (see legend). Error bars indicate the mean standard error. The right plot shows OE changes for male and female leaders. Significance is defined as follows: ns–not significant; * *p* < .1; ** *p* < .05; *** *p* < .001.

**Fig 2:**
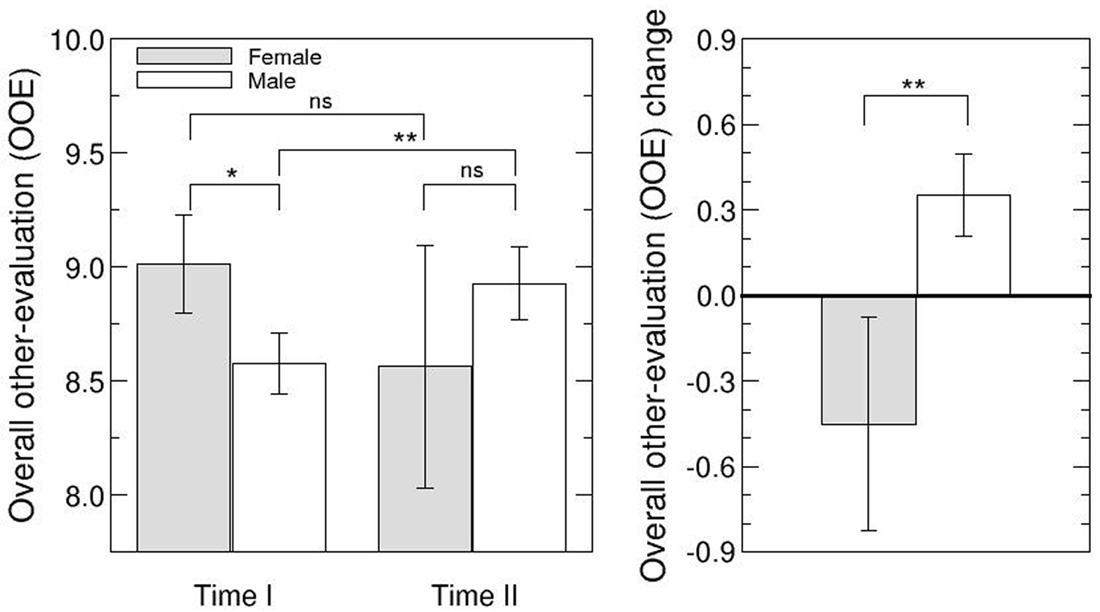
Analysis of overall other-evaluation (OOE). The left plot shows means for leaders’ OOE at Time I and Time II for male and female leaders (see legend). Error bars indicate the mean standard error. The right plot shows OOE changes for males and females. Significance is defined as follows: ns–not significant; * *p* < .1; ** *p* < .05; *** *p* < .001.

To analyze the *evaluations of leaders by team members (OE and OOE)* we included only those participants who completed the survey at Time I and Time II, to avoid sample bias and to ensure the highest level of reliability in the aggregated data. We analyzed responses of 223 team members from 44 teams (15 headed by a female leader; 29 by a male leader). In order to interpret the other-evaluation of female and male leaders, we compared the other-evaluation index (OE) of male and female leaders for both times of measurement. Team members evaluated female leaders more favorably than male leaders at Time I (*U* = 152.5, *p* = .055) (see Hypothesis 2), but the difference was below the conventional level of significance. At Time II, however, they evaluated female and male leaders similarly (*U* = 189.5, *p* = .248) (see left plot of Fig 1 and Table 2) (see Hypothesis 2). These findings were supported by the analysis of overall other-evaluations (OOE) (using the mean evaluation each leader obtained from all team members, *N* = 29, 11 female team members, 18 male team members) at both times of measurement. At Time I, female leaders tended to be evaluated more favorably than male leaders, *F*(1,28) = 3.32, *p* = .0799), but there was no gender difference at Time II (*U* = 89.0, *p* = .490) (see left plot of Fig 2 and Table 2) (see Hypothesis 2).

**Table 2:** Gender differences in other-evaluation index (OE), overall other-evaluation index (OOE) and relative clustering (RC) at Time I (TI) and Time II (TII).

Gender	OE TI	*SD*	OE TII	*SD*	OOE TI	*SD*	OOE TII	*SD*	RC TI	*SD*	RC TII	*SD*
**Female**	8.52	.35	8.23	.40	9.01	.22	8.56	.53	.92	.06	1.03	.13
**Male**	8.17	.18	8.58	.19	8.58	.13	8.93	.16	1.08	.08	.90	.11

Additionally, we investigated whether self-evaluations were correlated with team member evaluations at Time I and Time II, for female and male leaders separately. For male leaders there was no significant relation between self- and other-evaluations, neither at Time I nor at Time II, *r*(27) = .198; *p* = .36 for Time I and *r*(27) = .38, *p* = .66 for Time II. For female leaders there was a significant positive correlation between self- and other-evaluations at Time II, *r*(13) = .537, *p* < .048, but not at Time I, *r*(13) = .35, *p* = .21. These results are consistent with earlier findings, which indicate that women are generally more accurate in their self-ratings than men, so that there is higher agreement between self- and other-ratings for female managers than for male managers [88].

Furthermore, we investigated changes in the other-evaluation of female and male leaders over time. Changes in the other-evaluation of female and male leaders were significantly different (OE: *F(1*,*28)* = 4.64, *p* = .041 and OOE *U* = 48.0, *p* = .021). This is due to the fact that male leaders were evaluated better at Time II than at Time I (OE: *Z* = 80.0, *p* = .005, OOE: *t(17)* = -2.18, *p* = .027), while there were no changes in the evaluation of female leaders from Time I to Time II (OE: *Z* = 33.0, *p* = .153, OOE: *Z* = 13.0, *p* = .48) (see Hypothesis 2).

### Cohesiveness in the process of team development

To determine team cohesiveness we analyzed the structure of all teams. Specifically, we examined two aspects: (i) the overall cohesiveness of the team and (ii) the relative cohesiveness of the network around the leader (see Hypothesis 3). For this purpose, we did not use any existing software but wrote our own code in Python and then used the scipy.stats module to perform the hypothesis-testing analyses, and the networkx module to compute network metrics. As mentioned above, we constructed team networks for Times I and II, using the yes/no answers to the question: “Would you choose this person to work with you in a new team?” To ensure the highest level of reliability in the aggregated data, we included only team members who completed the survey at both times of measurement (223 team members, 44 teams, 15 female leaders, 29 male leaders).

**Fig 3:**
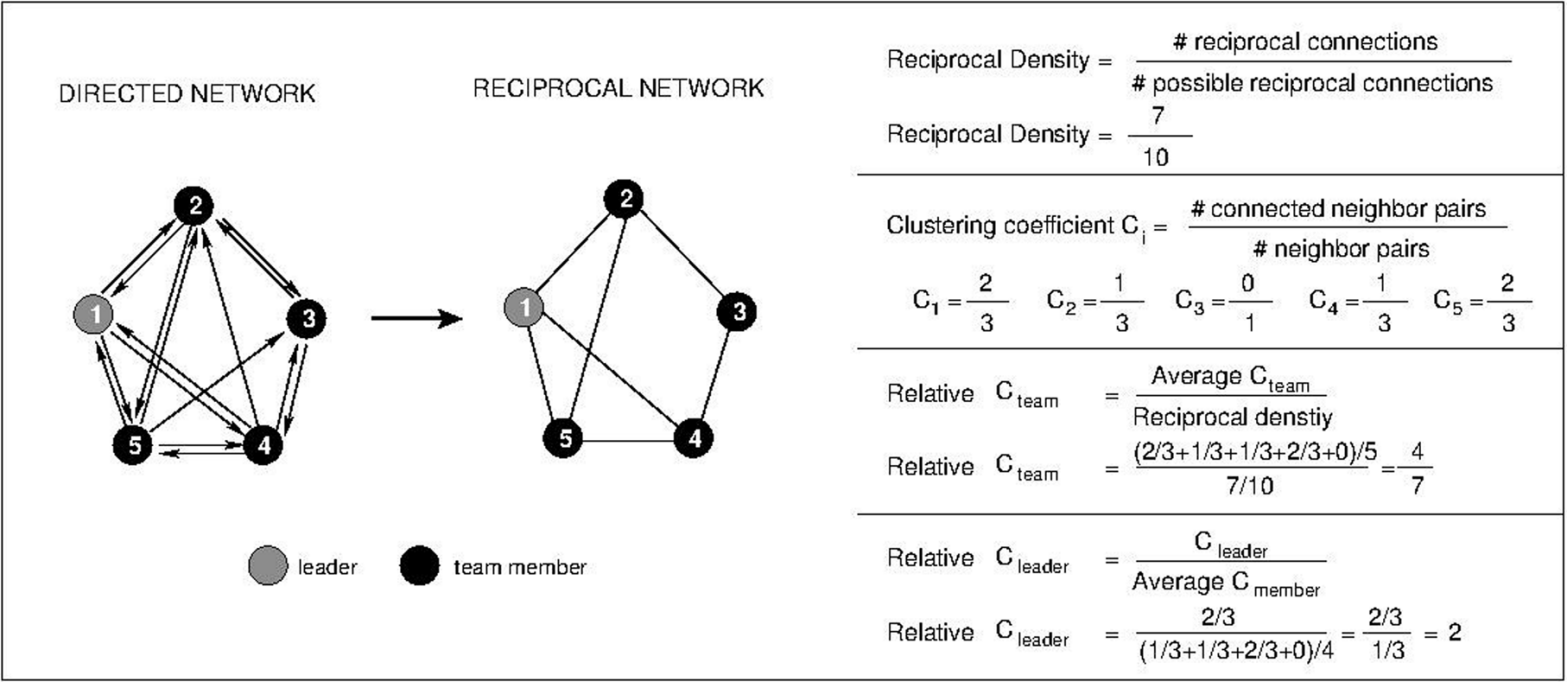
Illustration of social network analysis. As an example of a directed network of relationships between pairs of neighbors taken from the survey (left diagram), we show how to obtain the reciprocal network and different network metrics: reciprocal density, the local clustering coefficient C_i_ for each team member, the relative clustering coefficient of the team C_team_ and the relative clustering of the leader C_leader_. In the left diagram, the directed network shows only positive interactions (‘Yes’ answers in the survey). Black nodes mark regular team members, whereas the grey node indicates the team leader. If two team members A and B have positive interactions A -> B and B-> A in the directed network, then there is a reciprocal interaction between A and B (right diagram). From the reciprocal network of interactions, we computed the quantities of interest as indicated in the diagram. The symbol ‘#’ indicates total numbers.

As outlined earlier, team cohesiveness is captured in network terms via reciprocity and closure. Reciprocity is the degree to which pairs of team members, A and B, wish to work with one another. We therefore constructed a network of reciprocal connections for each team (see Fig 3). We connected the team members A and B if there was a connection from A to B and a connection from B to A in the directed network. To quantify reciprocity we computed the *reciprocal density*, i.e., the number of connected pairs out of all possible pairs of team members [89].

Closure in social networks refers to the existence of triangles in the social network of (reciprocal) interactions. Therefore, we computed the relative clustering coefficient of the team. More specifically, the clustering coefficient of team member A is the number of pairs of neighbors of A that are connected in the proportion of the team [90]. The clustering coefficient of the team is then computed as the average clustering coefficient of all team members. However, the larger the number of connections in a team, the larger is the clustering coefficient. Therefore, we needed to control for the density of connections to compare closure in different teams. To this end, we computed the *relative clustering coefficient of a team* (RCT), which is the average clustering coefficient divided by the reciprocal density (which equals the probability that two team members B and C that are connected with A are also connected with each other).

Interestingly, we found no differences in overall cohesiveness between female-led and male-led teams at Times I and II (Reciprocal density Time I: *U* = 137.5, *p* = .381; Time II: *U* = 143.5, *p* = .460; RCT Time I: *U* = 90.0, *p* = .012; Time II: *U* = 90.0, *p* = .151). Note that the significant differences at Time I are due to male-led teams having the same relative clustering coefficient of 1, which probably goes back to the low density of some of these teams; however, we believe that this is unlikely to be a general feature of male-led teams, which renders the difference not meaningful. In fact, the change in the relative clustering of teams was very small, both for male- and female-led teams, throughout our analysis (change for female leaders: *Z* = 3.0, *p* = 1.0; change for male leaders: *Z* = 12.0, *p* = .735).

**Fig 4:**
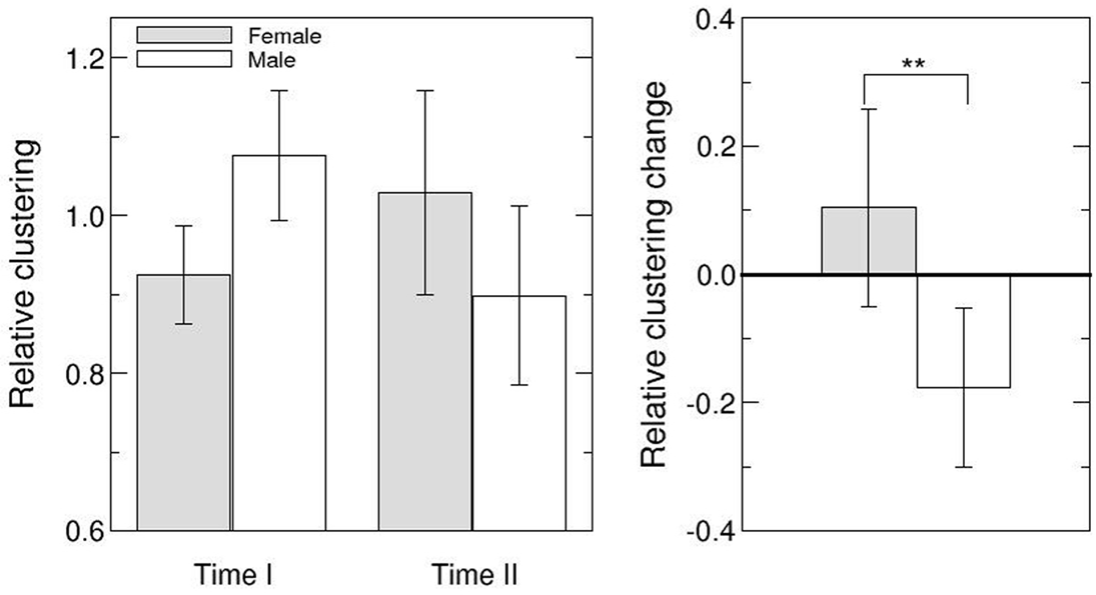
Analysis of the relative clustering (RC) around leaders. The left plot shows mean RCs at Time I and Time II for male and female leaders (see legend). Error bars indicate the mean standard error. The right plot shows changes in RC for males and females. Note that the change in leaders’ RC is significantly different for males and females (*p* < .05).

Next, we investigated the cohesiveness around each team leader in order to capture differences that might have emerged over time due to a possible link between leader gender and team cohesiveness. Note that investigating team cohesiveness around the leader amounts to determining the centrality of the leader within the team in terms of reciprocity and closure. The former aspect is based on the density of reciprocal leader-member connections and the latter on the clustering of network interactions around the leader, that is, the number of triangles in the network involving the leader. To assess the relative cohesiveness of the network of connections around the leader, we used the *relative clustering coefficient* (RC) of the leader, which is the ratio between the clustering coefficient of the leader *C*_*leader*_ and the *average clustering coefficient* of the remaining team members (see Fig 4). Note that if RC > 1, interactions cluster more around the leader than around other team members, whereas in case of RC<1 team interactions cluster more around other team members than the leader. We found a significant difference in the change of relative clustering coefficients for male and female leaders (*U* = 55.5, *p* = .032, cf. Fig 4). The RC of female leaders tended to increase from being lower than that of team members (RC < 1) to exceeding that of team members (RC > 1), while the RC for male leaders showed the opposite trend. Our results thus indicate that only female leaders build teams in which team member relationships increasingly cluster around the leader in the process of team development.

We did not find significant differences between male and female leaders in any other measure of leader centrality in the network, probably because these measures do not capture traits that are characteristic of leadership styles (Table 2).

## Discussion

The present research provides first insights into the impact of gender stereotypes on self-evaluations of female and male leaders, on their evaluation by team members and on cohesiveness in the processes of team development in a male-dominated field, namely engineering. The strength of the present study lies not only in its longitudinal design, but also in the fact that it was conducted in a well-structured setting that bears resemblance to an authentic professional setting. The teams worked on comparable tasks (creating a chemical plant) over the same period of time (nine months), leaders and team members possessed similar amounts of experience and had access to similar resources offered by the university. Such standardized conditions would be very difficult to find in other organizational settings, where leaders with different experience work on different projects with different time frames (even within the same department).

At the onset of the projects female leaders were expected to evaluate themselves less favorably than male leaders, due to the impact of gender stereotypes on women’s self-views [40]; this gender difference was assumed to disappear towards the end of the projects (Hypothesis 1). Contrary to our prediction, however, we did not find a gender difference in leaders’ self-evaluations at the start of the project. One possible explanation could be that in our sample all project leaders had to pass a fairly strict selection process to become leaders and were appointed by persons in charge of the program. Being selected and appointed as leader may have strengthened the self-evaluation of female leaders and thus leveled the self-evaluations of women and men. Interestingly, the self-evaluation of female leaders even surpassed that of male leaders at the end of the project. This could be explained with a gain in self-confidence on the part of women. In other words, leading a team successfully may have boosted female leaders’ self-esteem and led to the observed improvement in self-evaluation. However, this explanation is of a speculative nature and further research is needed to explain such shifts in female leaders’ self-views.

As for the evaluation of leaders by others, team members showed the expected tendency to evaluate female leaders better than male leaders at the start of their joint work (see Hypothesis 2), in accord with the shifting standards model [56,91], whereas evaluations of female and male leaders became similar at the end (Time II; see Hypothesis 2). These results document a certain female advantage at the beginning of the project and confirm earlier findings [92]. Unexpectedly, however, we observed that the evaluation of male leaders by team members improved at the end of the project. There may be at least two explanations for this improvement: On the one hand, male leaders match the stereotypical image of a leader better than female leaders [1,10]. Hence, team members might still evaluate male leaders more favorably, and this male advantage might cumulate and intensify over time. In this case individuation would not have occurred and the influence of the stereotype would have increased (rather than decreased; e.g., [91,93]) in the process of team development. On the other hand, male leaders may not only have been perceived differently but also may have behaved differently, for example, in a more agentic way than female leaders. In male-dominated fields such as engineering, team members may value and expect a more agentic leadership style (e.g., more goal-orientation) (e.g., [94]). Although male leaders were evaluated slightly less favorably at the beginning of the projects in the present research, they may have displayed the agentic behaviors consistent with the leader stereotype and may therefore have received an extra plus in the final evaluations (e.g., [14]). Anyway, female and male leaders were evaluated similarly at the end of the projects in the present study.

Finally, drawing on earlier research on gender differences in team cohesiveness (e.g., [71]), we expected female leaders to create teams that showed more cohesiveness at the end of the projects (Hypothesis 3). Indeed, we found a tendency for female leaders to get connections to cluster around them more than male leaders—although there were no differences in overall team cohesiveness between teams with male and female leaders. Our findings are in line with previous research [71] and suggest that female leaders may be more focused on building and sustaining relationships [95] and that they promote interaction styles that support and maintain social exchanges [96], which translates into a higher cohesiveness of teams led by female compared to male leaders.

Our findings have broader theoretical implications. So far, there has been little research on long-term changes in the perception of female and male leaders ([91] is an exception). Our study provides empirical evidence for existing theoretical positions by documenting that a female advantage in the evaluation of leaders decreased over time in a natural setting. Furthermore, we found that the teams supervised by female leaders were characterized by more connections between leaders and team members than the teams with male leaders. This may go back to a tendency of female leaders to emphasize collaboration and teamwork and to use a participative and interpersonally oriented leadership style (see the meta-analyses by [79,97,98]). A stronger connection between female leaders and their team members may be especially beneficial when groups have to solve socially complex tasks which, for example, require discussion, negotiation or coordination between functionally diverse or geographically dispersed units ([71]; see also [99,100]). Therefore, our study is a first step towards capturing gender differences in team network structures in a real-life setting. However, more research is needed to examine whether gender differences in team structure exist in other settings as well (e.g., organizational settings).

Moreover, future studies should take leaders’ self-construal in their theoretical reasoning into consideration. Nowadays self-evaluations of women and men are becoming more and more similar. For instance, female and male business students were found to ascribe themselves task- and person-oriented traits (agentic and communal traits, respectively) to a similar extent [101]. Therefore, the gender differences in leadership styles exhibited by women and men may have been smaller than expected, and capturing them with the help of network structure may have been difficult. Our assumption that different leadership styles are favored by female and male leaders and that they translate into team structure hence warrants more testing. For instance, previous studies have documented that cohesiveness translates into better team performance (e.g., [102,103]); theoretical explanations should therefore focus more on the conditions under which other- vs. task-orientation yields better communication and cohesiveness [71].

Finally, some practical implications of the study should be mentioned. First, female leaders’ self-evaluation was similar to male leaders’ self-evaluation at the start of the project and became more favorable in the course of the team development. This suggests that female leaders experienced a gain in self-confidence. Being appointed a leader by third parties (e.g., selection authorities) may not only have prevented women from evaluating themselves less favorably in the beginning, but may also have reduced the influence of gender stereotypes on women’s self-views as they developed their leader role. Second, our results show a certain advantage of female leaders at the onset of the projects. In line with the shifting standards model [56], team members seem to have measured the leadership competence of individual female leaders against lower standards of competence and that of individual male leaders against higher standards of competence. However, once female leaders had taken up their role as leaders, this tendency diminished.

Finally, our findings suggest that female leaders may be more effective in organizational settings requiring high networking skills of leaders. In teams with a history of conflict or communication issues, for example, the female leadership style might be more effective at preserving a close-knit core structure within the team that warrants its functioning.

### Limitations and future studies

The fact that our study was conducted in close collaboration with a public university enabled us to gather data in an ongoing university program over three academic years. However, it also implied some limitations. Firstly, the data relied on the pre-existing measures applied by the university. Future research should examine the role of time in self- and other-evaluations of leaders with the help of established scales (e.g., [104–107]). Secondly, while our study extends knowledge on the role of gender in the process of team development and points to potential underlying mechanisms, subsequent research should systematically investigate possible underlying mechanisms such as self-construal, gender group identification, or leadership styles. Thirdly, due to ethical regulations at the participating university we had no access to any objective measure of effectiveness, neither for the leaders nor for the teams (e.g., grades that leaders received for their work). Thus, we do not know whether leaders’ self-evaluation and their evaluation by team members or cohesiveness in the team were associated with a better outcome of the projects. Fourthly, due to the similarity in the gender composition of all teams (i.e., slightly more males) and the relatively small sample, we were not able to analyze the impact of the teams’ gender composition on the self- and other-evaluation of leaders and on team cohesiveness. Such an impact can be expected, because the little research there indicates differences in how female and male subordinates evaluate female and male leaders (e.g., [50,108]), with men being more likely than women to attribute successful manager characteristics to women [109]. Previous findings also suggest that gender diversity can have negative (e.g., [110,111]) as well as positive effects (e.g., [112–114]) on team processes and outcomes.

### Conclusions

The present research indicates that team leaders’ gender plays a role for certain evaluations: In our study, female leaders tended to evaluate themselves more favorably at the end of a project than at the beginning, and their self-evaluations were more favorable than those of men at the end. Team members tended to evaluate female leaders better than male leaders at the onset of the collaboration. In the process of team development, however, this tendency faded away. At a later stage, leaders’ evaluations by team members seemed to be determined by individuation rather than gender stereotyping [23,26,59]. In addition, networks headed by women showed a slightly higher cohesiveness than those headed by men. These findings expand our knowledge on stereotypical biases in the perception of female and male leaders: We were able to demonstrate that the influence of stereotypes on self- and other-evaluations of leaders changes in the process of team development. Analyzing network structure, we also showed that female leaders, even in a highly male-dominated context, may be more successful in building teams that are more cohesive.

## Supporting information

S1 Dataset Raw data obtained from the responses of leader evaluations by other team members (spreadsheet 1) and from the self-evaluation survey of the leaders (spreadsheet 2). Spreadsheet 3 contains the dictionary of the data.
(XLS)
